# Evaluating Super-Resolution Models in Biomedical Imaging: Applications and Performance in Segmentation and Classification

**DOI:** 10.3390/jimaging11040104

**Published:** 2025-03-29

**Authors:** Mario Amoros, Manuel Curado, Jose F. Vicent

**Affiliations:** Department of Computer Science and Artificial Intelligence, University of Alicante, Campus de San Vicente del Raspeig, Ap. Correos 99, E-03080 Alicante, Spainjvicent@ua.es (J.F.V.)

**Keywords:** super-resolution, segmentation, classification

## Abstract

Super-resolution (SR) techniques have gained traction in biomedical imaging for their ability to enhance image quality. However, it remains unclear whether these improvements translate into better performance in clinical tasks. In this study, we provide a comprehensive evaluation of state-of-the-art SR models—including CNN- and Transformer-based architectures—by assessing not only visual quality metrics (PSNR and SSIM) but also their downstream impact on segmentation and classification performance for lung CT scans. Using U-Net and ResNet architectures, we quantify how SR influences diagnostic tasks across different datasets, and we evaluate model generalization in cross-domain settings. Our findings show that advanced SR models such as SwinIR preserve diagnostic features effectively and, when appropriately applied, can enhance or maintain clinical performance even in low-resolution contexts. This work bridges the gap between image quality enhancement and practical clinical utility, providing actionable insights for integrating SR into real-world biomedical imaging workflows.

## 1. Introduction

Medical imaging has become an indispensable tool in modern healthcare, playing a pivotal role in diagnosis, monitoring, and treatment planning for a wide range of diseases. Focusing on the lungs, conditions such as pneumonia, cancer, and chronic obstructive pulmonary disease (COPD) represent significant health challenges [1,2]. Technologies such as Computed Tomography (CT) scanning are widely used for visualizing anatomical structures and identifying pathologies, offering high spatial resolution and three-dimensional visualization capabilities that enhance diagnostic accuracy [3]. However, despite continuous advancements, challenges remain in detecting subtle or early-stage abnormalities, such as small nodules or infiltrative lesions, which is crucial for timely diagnosis and effective treatment planning [4].

One of the primary limitations of conventional medical imaging lies in its resolution constraints. CT images often suffer from limited spatial resolution, which can hinder the accurate detection of small pathological features, such as early-stage tumors or micro-lesions [5,6]. This limitation can result in misclassification or missed diagnoses, especially in lung cancer detection, where early intervention is crucial [7,8]. Thus, enhancing image resolution has become a focal point in medical imaging research to improve the visualization of fine details necessary for accurate diagnosis [9].

Super-resolution (SR) techniques have emerged as a promising solution to enhance the spatial resolution of medical images. Traditional methods, such as interpolation-based techniques, often produce smooth images that lack fine texture details [10]. More recently, deep learning-based approaches, particularly those using Convolutional Neural Networks (CNNs) and Generative Adversarial Networks (GANs), have shown significant improvements in generating high-resolution images that reveal diagnostic details not visible in lower-resolution inputs [11,12]. For instance, CNN-based SR models have demonstrated effectiveness in enhancing the resolution of chest CT scans, improving the detection of small nodules indicative of early-stage lung cancer.

In the context of medical imaging, combining SR with deep learning models, such as multi-head convolutional attention networks, has shown promising results in enhancing the quality of MRI and CT images. These advancements not only improve classification and segmentation accuracy but also support other applications, such as image-guided surgery and real-time diagnostic procedures [13,14]. By leveraging SR techniques, it is possible to reduce noise in low-dose CT scans while preserving critical features, thereby enhancing diagnostic confidence while minimizing patient exposure to radiation.

In this study, we aim to systematically evaluate the impact of various deep learning-based SR techniques on the performance of segmentation and classification models in medical imaging. By comparing models trained on super-resolved images and those trained on original high-resolution scans, we seek to determine whether SR truly enhances diagnostic performance or merely maintains existing standards [15]. We will explore state-of-the-art methods, including CNNs, GANs, and hybrid architectures, to assess their efficacy in optimizing medical imaging workflows and improving patient outcomes [16].

## 2. Related Work

Super-resolution (SR) algorithms have traditionally been classified into three main categories: interpolation-based methods [17], reconstruction-based methods, and learning-based methods [18]. Interpolation methods estimate pixel values to generate high-resolution (HR) images from low-resolution (LR) inputs. Although computationally efficient, they often fail to recover fine details, resulting in blurred outputs. Reconstruction-based approaches incorporate prior knowledge about the imaging process to better constrain the SR problem; while more accurate, these methods are typically computationally intensive. Learning-based methods—especially those leveraging deep learning—have transformed the field by enabling complex mappings from LR to HR images.

### 2.1. Deep Learning-Based Super-Resolution Techniques

The advent of deep learning has fundamentally reshaped SR research. Dong et al. [19] introduced SRCNN, the first convolutional neural network (CNN)-based model for SR, proving that end-to-end learning can significantly outperform traditional approaches. Building upon this, EDSR introduced deeper architectures with residual connections, leading to substantial gains in both image quality and training efficiency.

Generative Adversarial Networks (GANs) marked a further breakthrough in SR. Ledig et al. [11] presented SRGAN, combining adversarial and perceptual losses to produce visually realistic textures. ESRGAN [20] refined this approach using Residual-in-Residual Dense Blocks (RRDB) and a relativistic discriminator, setting a new benchmark in perceptual image quality.

More recently, Transformer-based models have emerged as a powerful alternative. SwinIR [21] employs the Swin Transformer to capture long-range dependencies, achieving state-of-the-art results in SR and other image restoration tasks. This evolution towards attention-based mechanisms mirrors broader trends in computer vision.

### 2.2. Super-Resolution in Medical Imaging

SR techniques are especially valuable in medical imaging, where high-resolution scans from modalities such as magnetic resonance imaging (MRI), computed tomography (CT), and X-rays are crucial for diagnosis and treatment planning.

Early efforts in medical SR employed CNN-based models. Umehara et al. [4] applied a CNN to chest CT images, achieving improvements in the Peak Signal-to-Noise Ratio (PSNR) and Structural Similarity Index (SSIM). However, these models were often trained on synthetically degraded images, limiting their real-world applicability.

GAN-based methods have shown promise in overcoming these limitations. Xu et al. [22] used GANs to enhance low-dose chest X-ray images, improving perceptual quality without compromising diagnostic relevance. Zhu et al. [23] introduced CSRGAN, a conditional GAN tailored for medical SR, producing clinically meaningful outputs guided by auxiliary information.

Transformer-based models, although relatively new to this domain, are beginning to show potential. SwinIR [21], for instance, has demonstrated efficacy in handling complex anatomical structures in MRI and CT scans, thanks to its ability to model long-range interactions while preserving diagnostic features.

### 2.3. Evaluation Metrics for Super-Resolution

The evaluation of SR methods typically relies on quantitative metrics such as PSNR, SSIM, and the Feature Similarity Index (FSIM) [24]. PSNR measures the fidelity of reconstructed images, while SSIM evaluates structural similarity, making it more aligned with human perception. FSIM focuses on image features such as phase congruency and gradient magnitude, providing additional insights into image quality.

In medical imaging, task-specific metrics are increasingly being used to evaluate SR performance. Some examples are as follows:

- Segmentation accuracy measures how well SR-enhanced images improve the performance of downstream segmentation tasks, often evaluated using the Dice coefficient or Intersection over Union (IoU) [25].
- Classification metrics assess the impact of SR on diagnostic accuracy; commonly used metrics include area under the curve (AUC) and F1 score [26].

These task-based evaluations provide a more holistic understanding of SR methods’ impact, particularly in applications where clinical utility is paramount.

## 3. Methodology

In this study, we propose a novel approach to evaluating super-resolution models applied to medical imaging, with a focus not only on enhancing image quality but also on assessing the impact of this improvement on downstream clinical tasks. Traditional evaluations of super-resolution models primarily focus on metrics such as PSNR or SSIM to assess image fidelity. However, these metrics alone do not fully capture whether improved resolution translates into better performance in real-world clinical applications.

To bridge this gap, we evaluate super-resolution models based on their effectiveness in critical medical applications, specifically targeting classification and segmentation tasks. For this purpose, we utilize lung CT scans to detect and segment various pulmonary diseases. Our approach aims to demonstrate that enhanced image resolution not only improves visual quality but also contributes to increased accuracy and reliability in diagnostic models.

By integrating super-resolution techniques into downstream tasks such as disease detection and segmentation, this study aims to bridge the gap between theoretical advancements in image quality and their practical utility in the medical field. This framework thus combines image quality and clinical relevance, contributing to the development of AI-driven diagnostic tools in healthcare.

**Figure 1:**
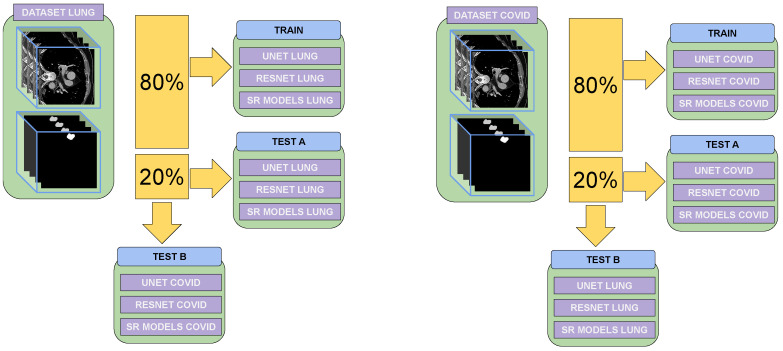
The evaluation and training processes using the two training datasets are presented. The figure illustrates how super-resolution models trained for one specific disease were tested for their ability to perform super-resolution for a different disease. The evaluation assesses how effectively these models contribute to classification and segmentation tasks tailored to the target disease.

As shown in Figure 1, two evaluations were conducted. The first evaluated the model’s ability to generate high-resolution images using the dataset on which it was trained, assessing its performance in this specific scenario. The second evaluation involved applying the model to an image from a different dataset, one on which it has not been trained, to analyze how well it would generalize to unseen cases and perform super-resolution on new data.

### 3.1. Super-Resolution Networks

In this study, we used several state-of-the-art super-resolution networks to improve the resolution and quality of medical images, specifically focusing on lung CT scans. The selected networks include the following:

- SRCNN (Super-Resolution Convolutional Neural Network): One of the pioneering models for super-resolution, SRCNN utilizes a shallow convolutional architecture to learn end-to-end mappings from low-resolution to high-resolution images. Despite its simplicity, SRCNN demonstrates significant improvements in image quality, making it a foundational model in the field [27].
- EDSR (Enhanced Deep Residual Networks for Single Image Super-Resolution): EDSR, an extension of the ResNet architecture, optimizes super-resolution tasks by removing batch normalization layers and enhancing the performance of residual blocks. This model achieves higher PSNR and SSIM scores than traditional residual networks, making it particularly effective at preserving fine details in medical images [2].
- SRResNet: Introduced alongside the SRGAN framework, SRResNet is designed to improve image resolution using deep residual blocks. This model focuses on enhancing structural details without introducing artifacts, proving effective in medical imaging where the preservation of structural integrity is crucial [11].
- RCAN (Residual Channel Attention Network): RCAN utilizes channel attention mechanisms to adaptively rescale feature maps, enhancing important details while suppressing less relevant information. This approach is particularly useful in medical imaging, where fine-grained details can be critical for diagnosis [12].
- SwinIR (Swin Transformer for Image Restoration): SwinIR leverages the Swin Transformer architecture, employing window-based attention mechanisms to capture both local and global features. This model excels in enhancing resolution while maintaining computational efficiency, making it a suitable choice for high-resolution medical imaging tasks [21].

These models were chosen for their proven effectiveness in super-resolution tasks, ranging from foundational methods such as SRCNN to more advanced approaches such as SwinIR, which excel at preserving fine details. This level of detail is especially important when evaluating downstream tasks such as classification and segmentation in medical imaging. Diffusion models were not included due to their high computational cost and the added complexity they introduce, which can be impractical for clinical applications requiring efficiency and clarity. These networks were evaluated based on their ability to not only improve image resolution but also enhance the performance of downstream tasks such as classification and segmentation of lung diseases, as is represented in Figure 1.

### 3.2. Evaluation of Classification

The high-resolution images were subsequently used to train a classification model to detect pulmonary diseases. We utilized a ResNet-50 architecture pre-trained on ImageNet, fine-tuned for this specific task. Metrics such as accuracy, F1-score, and precision were used to evaluate the performance of the classification model. Then, we used this network for evaluate the super-resolution images from each network.

### 3.3. Evaluation of Segmentation

For the segmentation task, a U-Net architecture was employed to delineate lung structures and detect disease regions in the CT scans. The Dice coefficient and Intersection over Union (IoU) were used to assess the quality of the segmentation, with particular focus on how super-resolution impacts these metrics.

## 4. Experiments

### 4.1. Datasets

For the experiments, two publicly available lung CT datasets were used: the Task06-Lung Dataset and a COVID-19 CT Dataset. Both datasets contain annotated 3D CT scan volumes and were utilized for segmentation and radiological analysis.

**Task06-Lung Dataset:** This dataset consists of 96 segmented 3D CT scan volumes of patients diagnosed with non-small cell lung cancer (NSCLC), collected at Stanford University, Palo Alto, CA, USA. It was made publicly available via TCIA [7,8,9] and has been used to develop radiogenomic signatures. The dataset is split into 64 training volumes and 32 test volumes. Each volume features a resolution consistent with thin-section CT protocols: slice thickness less than 1.5 mm, tube voltage 120 kVp, automated tube current modulation (100–700 mA), tube rotation speed of 0.5 s, helical pitch between 0.9 and 1.0, and a sharp reconstruction kernel. Tumor regions were manually annotated by a thoracic radiologist using dedicated medical imaging software.

**Figure 2:**
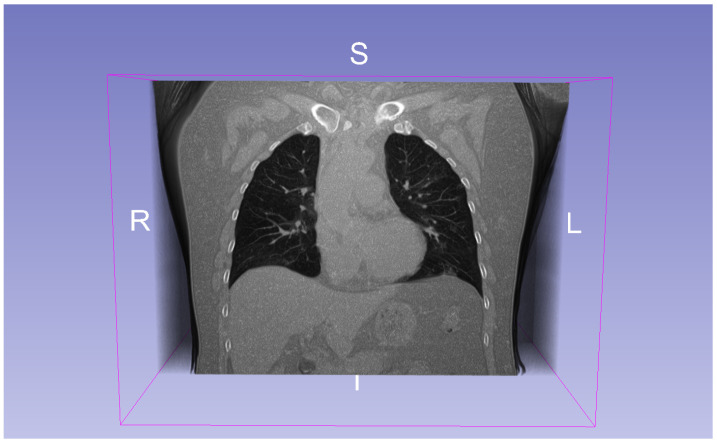
A CT scanfrom the COVID-19 dataset. R indicates the right side, L the left side, I the inferior direction, and S the superior direction in the coordinate space.

**COVID-19 CT Dataset:** This dataset comprises 20 annotated 3D CT scan volumes of patients diagnosed with COVID-19 [28]. Each volume has a standardized shape of 256×256×256 voxels. The dataset includes segmentations for the left lung, right lung, and infection regions (see Figure 2). Two radiologists independently performed the annotations, which were later reviewed and validated by a senior radiologist. The CT scans in this dataset were compiled from various sources, including COVID-19-specific scans, labeled non-COVID lung CT scans, and heterogeneous datasets. This diversity was used to improve annotation quality and robustness in segmentation tasks.

### 4.2. Image Preprocessing

For image preprocessing, all CT scans underwent a cropping process to extract a Region of Interest (ROI). This technique focused on the most relevant areas for identifying clinical issues, particularly targeting the pulmonary region where the main objectives are located.

The cropping process involved extracting a cubic volume of 256×256×256 pixels. In the x and y dimensions, the 256 most central points were selected to ensure that the area of interest, typically located near the image’s center, was entirely included within the cropped volume. For the z dimension, corresponding to axial slices, the most central slices were chosen. If the available number of slices was insufficient to reach 256, black images were added to meet the required dimensions. This cropping approach offers several advantages. First, it reduces the quantity of data to be processed, decreasing computation time and storage requirements. Second, by focusing on a smaller and more relevant region, it enhances the accuracy of super-resolution, classification, and segmentation models by removing irrelevant information that might confuse the model. Once the images had been cropped and the ROI had been extracted, advanced image processing techniques were applied to improve their quality and resolution. These techniques included super-resolution to transform low-resolution images into high-resolution ones and segmentation to accurately identify and delineate pulmonary structures and disease-affected areas.

Finally, the resolution of the processed images was reduced fourfold to achieve a resolution of 64×64. This reduced-resolution dataset served as the input to the network.

### 4.3. Tests Performed

This section presents the various tests conducted to evaluate the quality of several super-resolution models. These tests can be categorized based on the nature of the evaluation as follows:

- Visual Metrics: These metrics assess image quality using indicators such as PSNR and SSIM. PSNR measures the ratio between the maximum possible power of a signal and the power of noise affecting its representation, providing an objective assessment of the fidelity of the reconstructed image. SSIM, on the other hand, evaluates the structural similarity between the original and reconstructed images, focusing on luminance, contrast, and structure.
- Functional Metrics: These metrics focus on improvements or error ratios in specific tasks, such as segmentation and classification. For example, they evaluate whether super-resolution enhances accuracy in delineating regions of interest in segmentation tasks or improves classification predictions for disease diagnosis.

The evaluation process incorporated two key functionalities for each type of metric:

1. Training and Testing on the Same Dataset: The model was trained on a specific dataset and tested on images from the same dataset. This approach helped to determine how well the model generalized within a controlled environment and served as a baseline for comparison.
2. Training and Testing on Different Datasets: The model was trained on one dataset and tested on a different dataset. This evaluated the model’s robustness and its ability to generalize across diverse data distributions, which are crucial for real-world applications.

These tests provided a comprehensive understanding of the strengths and limitations of each super-resolution model. By analyzing both visual and functional metrics across different training and testing scenarios, the evaluation ensures that the selected models meet the necessary standards for clinical and research applications.

## 5. Results

### 5.1. Segmentation Results

In the segmentation phase, super-resolution was applied to the test set images using all networks, regardless of the dataset on which they were trained. These enhanced images were then processed by a pre-trained segmentation network. This approach allowed us to evaluate how resolution improvement influences segmentation accuracy.

**Figure 3:**
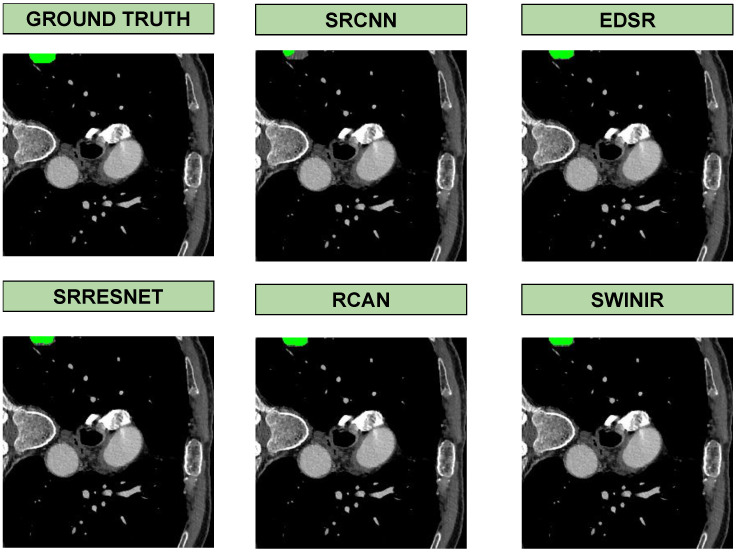
Visual results of segmentation in the output of each super-resolution method on the COVID dataset.

Based on the segmentation results in Figure 3, the quality of super-resolution directly impacts segmentation accuracy. SwinIR, being the most powerful model, produced enhanced images that led to segmentation outputs closely matching the ground truth. In contrast, SRCNN, as the most basic model, resulted in less accurate segmentations, likely due to the loss of fine details and lower reconstruction quality. Models such as RCAN and EDSR also showed competitive results, but SwinIR stood out in preserving structural details crucial for segmentation. This highlights the importance of using high-quality super-resolution models to improve downstream medical image analysis tasks.

The results shown in Table 1 indicate that models generally performed better when tested on the same issue on which they were trained. For instance, when trained and tested on COVID images, SwinIR achieved the highest Dice score (0.8054) and IoU score (0.6647), while SRCNN performed best when trained and tested on Task06-Lung images (Dice score: 0.7468; IoU score: 0.6547). However, when models trained on Task06-Lung images were tested on COVID data, their performance dropped, suggesting a limited ability to generalize across datasets. Conversely, models trained on COVID images and tested on Task06-Lung data maintained more stable performance, indicating that COVID-trained models may generalize better. Among all models, SwinIR consistently delivered the best results, while SRCNN performed the worst, highlighting the superiority of more advanced architectures such as SwinIR, EDSR, and SRResNet. These findings emphasize the impact of training data on super-resolution model performance and suggest that more diverse datasets contribute to better generalization.

### 5.2. Classification Results

In the classification phase, super-resolution was applied to the test set images using all networks, regardless of the dataset on which they were trained. These enhanced images were then processed by a pre-trained segmentation network. This approach allowed us to evaluate how resolution improvement influences classification accuracy (Table 2).

## 6. Discussion and Conclusions

The comprehensive evaluation of super-resolution networks revealed distinct performance characteristics across different architectures. SRCNN, as the foundational model, demonstrated the most limited capabilities, consistently showing the lowest performance in both segmentation and classification tasks. In contrast, more advanced networks such as EDSR, SRResNet, RCAN, and SwinIR exhibited progressively improved results, highlighting the significant advancements in deep learning-based image enhancement techniques. SwinIR emerged as the standout performer, leveraging Transformer-based architecture to capture intricate details and maintain structural integrity across medical imaging datasets. EDSR showed remarkable precision, particularly in COVID-19 image processing, while SRResNet delivered consistent performance across different scenarios. RCAN, with its channel attention mechanism, provided a balanced approach to image resolution, effectively preserving important diagnostic features. Each network demonstrated unique strengths: SwinIR excelled in overall performance, EDSR in precision, SRResNet in generalizability, and RCAN in feature preservation. The research underscores the critical challenges in medical image super-resolution, particularly in cross-dataset generalization; while these advanced networks show promising results, significant limitations remain in creating universally applicable super-resolution models. Future research should focus on developing more robust architectures that can maintain performance across diverse medical imaging modalities, potentially through hybrid approaches or more comprehensive training strategies. The ultimate goal remains to create super-resolution techniques that can consistently enhance diagnostic capabilities across various medical imaging scenarios. Clinically, the application of high-performing super-resolution models—particularly SwinIR and EDSR—has the potential to reduce the need for repeated imaging procedures, lower exposure to radiation in modalities such as CT, and enhance diagnostic accuracy in resource-limited settings where high-resolution imaging may not be available. Moreover, improved image quality may aid radiologists in detecting subtle pathological features, contributing to faster and more reliable diagnoses in diseases such as COVID-19, cancer, and neurological disorders.

Future research should explore the integration of these super-resolution models into real-world clinical workflows, evaluating their impact on diagnostic decision-making, inter-observer variability, and patient outcomes. In conclusion, super-resolution techniques offer a transformative approach to medical imaging, enabling the enhancement of low-quality images to reveal critical diagnostic details. By leveraging advanced deep learning models, these methods promise to improve early disease detection and support more precise medical assessments.

## Figures and Tables

**Table 1:** Dice and IoU scores of segmentation models. In bold and underline, the best results for each metric.

Model	Training Data	Test Data	Dice Score	IoU Score
**No Super-Resolution**	–	COVID	0.6921	0.5276
SRCNN	Task06-Lung	COVID	0.5551	0.4076
EDSR	Task06-Lung	COVID	0.6485	0.4817
SRResNet	Task06-Lung	COVID	0.6710	0.5063
RCAN	Task06-Lung	COVID	0.6248	0.4560
SwinIR	Task06-Lung	COVID	**0.6856**	**0.5260**
SRCNN	COVID	COVID	0.6703	0.4677
EDSR	COVID	COVID	0.7220	0.6171
SRResNet	COVID	COVID	0.7115	0.6295
RCAN	COVID	COVID	0.6328	0.4642
SwinIR	COVID	COVID	**0.8054**	**0.6647**
**No Super-Resolution**	–	Task06-Lung	0.7121	0.5966
SRCNN	Task06-Lung	Task06-Lung	**0.7308**	**0.6371**
EDSR	Task06-Lung	Task06-Lung	0.6888	0.5935
SRResNet	Task06-Lung	Task06-Lung	0.6797	0.5880
RCAN	Task06-Lung	Task06-Lung	0.6454	0.5562
SwinIR	Task06-Lung	Task06-Lung	0.6921	0.6001
SRCNN	COVID	Task06-Lung	0.7343	0.6415
EDSR	COVID	Task06-Lung	**0.7497**	0.6533
SRResNet	COVID	Task06-Lung	0.7468	**0.6547**
RCAN	COVID	Task06-Lung	0.6812	0.5905
SwinIR	COVID	Task06-Lung	0.7017	0.6062

**Table 2:** Classification metrics of models. In bold and underline, the best results for each metric.

Model	Training Data	Test Data	Accuracy	Precision	Recall	F1 Score
**No Super-Resolution**	–	COVID	0.9509	0.9643	0.9024	0.9621
SRCNN	Task06-Lung	COVID	0.9109	0.9633	0.8824	0.9211
EDSR	Task06-Lung	COVID	**0.9554**	**0.9825**	0.9412	**0.9614**
SRResNet	Task06-Lung	COVID	0.9406	0.9652	0.9328	0.9487
RCAN	Task06-Lung	COVID	0.9505	0.9739	0.9412	0.9573
SwinIR	Task06-Lung	COVID	0.9406	0.9421	**0.9580**	0.9500
SRCNN	COVID	COVID	0.9109	0.9633	0.8824	0.9211
EDSR	COVID	COVID	**0.9554**	**0.9825**	0.9412	**0.9614**
SRResNet	COVID	COVID	0.9406	0.9652	0.9328	0.9487
RCAN	COVID	COVID	0.9505	0.9739	0.9412	0.9573
SwinIR	COVID	COVID	0.9406	0.9421	**0.9580**	0.9500
**No Super-Resolution**	–	Task06-Lung	0.9589	0.9621	0.6421	0.9621
SRCNN	Task06-Lung	Task06-Lung	0.9525	0.9677	0.5263	0.7518
EDSR	Task06-Lung	Task06-Lung	0.9593	0.9459	0.6140	0.7447
SRResNet	Task06-Lung	Task06-Lung	0.9542	0.9167	0.5789	0.7097
RCAN	Task06-Lung	Task06-Lung	0.9593	0.8837	**0.6667**	0.7600
SwinIR	Task06-Lung	Task06-Lung	**0.9610**	**0.9677**	**0.6667**	**0.7677**
SRCNN	COVID	Task06-Lung	0.9559	**1.0000**	0.5439	**0.7045**
EDSR	COVID	Task06-Lung	0.9168	**1.0000**	0.1404	0.2462
SRResNet	COVID	Task06-Lung	0.9100	**1.0000**	0.0702	0.1311
RCAN	COVID	Task06-Lung	0.9338	0.6406	**0.7193**	0.6777
SwinIR	COVID	Task06-Lung	**0.9525**	0.9677	0.5263	0.6818

## Data Availability

The original data presented in the study are openly available at https://github.com/Maristoteles02/CT-RefineNet.

## References

[1] Izonin I., Tkachenko R., Peleshko D., Rak T., Batyuk D. Learning-Based Image Super-Resolution Using Weight Coefficients of Synaptic Connections. Proceedings of the 2015 Xth International Scientific and Technical Conference on Computer Sciences and Information Technologies (CSIT).

[2] Lim B., Son S., Kim H., Nah S., Lee K. Enhanced deep residual networks for single image super-resolution. Proceedings of the IEEE Conference on Computer Vision and Pattern Recognition Workshops (CVPRW).

[3] Li R., Xiao C., Huang Y., Hassan H., Huang B. (2022). Deep Learning Applications in Computed Tomography Images for Pulmonary Nodule Detection and Diagnosis: A Review. Diagnostics.

[4] Umehara K., Ota J., Ishida T. (2018). Application of Super-Resolution Convolutional Neural Network for Enhancing Image Resolution in Chest CT. J. Digit. Imaging.

[5] Georgescu M.I., Ionescu R., Miron A.I., Savencu O., Ristea N.C., Verga N., Khan F. (2022). Multimodal Multi-Head Convolutional Attention with Various Kernel Sizes for Medical Image Super-Resolution. arXiv.

[6] Li Y., Sixou B., Peyrin F. (2021). A Review of the Deep Learning Methods for Medical Images Super Resolution Problems. IRBM.

[7] Gevaert O., Xu J., Hoang C., Leung A., Xu Y., Quon A., Rubin D., Napel S., Plevritis S. (2012). Non-Small Cell Lung Cancer: Identifying Prognostic Imaging Biomarkers by Leveraging Public Gene Expression Microarray Data–Methods and Preliminary Results. Radiology.

[8] Napel S. (2014). NSCLC Radiogenomics: Initial Stanford Study of 26 Cases.

[9] Bakr S., Gevaert O., Echegaray S., Ayers K., Zhou M., Shafiq M., Zheng H., Benson J., Zhang W., Leung A. (2018). A Radiogenomic Dataset of Non-Small Cell Lung Cancer. Sci. Data.

[10] Horé A., Ziou D. Image Quality Metrics: PSNR vs. SSIM. In Proceedings of the 2010 20th International Conference on Pattern Recognition.

[11] Ledig C., Theis L., Huszár F., Caballero J., Cunningham A., Acosta A., Aitken A., Tejani A., Totz J., Wang Z. Photo-Realistic Single Image Super-Resolution Using a Generative Adversarial Network. Proceedings of the IEEE Conference on Computer Vision and Pattern Recognition.

[12] Zhang Y., Li K., Li K., Wang L., Zhong B., Fu Y. Image Super-Resolution Using Very Deep Residual Channel Attention Networks. Proceedings of the European Conference on Computer Vision (ECCV).

[13] Deka B., Datta S., Mullah H., Hazarika S. (2020). Diffusion-weighted and spectroscopic MRI super-resolution using sparse representations. Biomed. Signal Process. Control.

[14] Amoros M., Curado M., Vicent J.F. CTextureFusion: Advanced Texture Transfer with Multi-head Attention for Improving Lung CT Super Resolution. Proceedings of International Conference on Pattern Recognition 2024.

[15] Wang Z., Chen J., Hoi S. (2021). Deep Learning for Image Super-Resolution: A Survey. IEEE Trans. Pattern Anal. Mach. Intell..

[16] Zhang Z., Wang Z., Lin Z., Qi H. (2019). Image Super-Resolution by Neural Texture Transfer. arXiv.

[17] Xiang R., Yang H., Yan Z., Mohamed Taha A., Xu X., Wu T. (2022). Super-Resolution Reconstruction of GOSAT CO_2_ Products Using Bicubic Interpolation. Geocarto Int..

[18] Higaki T., Nakamura Y., Tatsugami F., Nakaura T., Awai K. (2019). Improvement of Image Quality at CT and MRI Using Deep Learning. Jpn. J. Radiol..

[19] Dong C., Loy C., He K., Tang X. Learning a Deep Convolutional Network for Image Super-Resolution. Proceedings of the Computer Vision—ECCV 2014.

[20] Wang X., Yu K., Wu S., Gu J., Liu Y., Dong C., Loy C., Qiao Y., Tang X. ESRGAN: Enhanced Super-Resolution Generative Adversarial Networks. https://github.com/xinntao/ESRGAN.

[21] Liang J., Cao J., Sun G., Zhang K., Van Gool L., Timofte R. SwinIR: Image Restoration Using Swin Transformer. Proceedings of the IEEE International Conference on Computer Vision Workshops (ICCVW).

[22] Xu L., Zeng X., Huang Z., Li W., Zhang H. (2020). Low-Dose Chest X-Ray Image Super-Resolution Using Generative Adversarial Nets with Spectral Normalization. Biomed. Signal Process. Control.

[23] Zhu Y., Zhou Z., Liao G., Yuan K. Csrgan: Medical Image Super-Resolution Using a Generative Adversarial Network. Proceedings of the ISBI Workshops 2020—International Symposium on Biomedical Imaging Workshops.

[24] Zhang L., Zhang L., Mou X., Zhang D. (2011). FSIM: A Feature Similarity Index for Image Quality Assessment. IEEE Trans. Image Process..

[25] Chen Z., Pawar K., Ekanayake M., Pain C., Zhong S., Egan G. (2022). Deep Learning for Image Enhancement and Correction in Magnetic Resonance Imaging—State-of-the-Art and Challenges. J. Digit. Imaging.

[26] Loizidou K., Elia R., Pitris C. (2023). Computer-Aided Breast Cancer Detection and Classification in Mammography: A Comprehensive Review. Comput. Biol. Med..

[27] Dong C., Loy C., He K., Tang X. Image Super-Resolution Using Deep Convolutional Networks. http://mmlab.ie.cuhk.edu.hk/.

[28] Ma J., Ge C., Wang Y., An X., Gao J., Yu Z., Zhang M., Liu X., Deng X., Cao S. (2020). COVID-19 CT Lung and Infection Segmentation Dataset.

